# Discovering cryptic pocket opening and binding of a stimulant derivative in a vestibular site of the 5-HT_3A_ receptor

**DOI:** 10.1126/sciadv.adr0797

**Published:** 2025-04-11

**Authors:** Nandan Haloi, Emelia Karlsson, Marc Delarue, Rebecca J. Howard, Erik Lindahl

**Affiliations:** ^1^ SciLifeLab, Department of Applied Physics, KTH Royal Institute of Technology, Tomtebodävagen 23, Solna, 17165 Stockholm, Sweden.; ^2^ SciLifeLab, Department of Biochemistry and Biophysics, Stockholm University, Tomtebodavägen 23, Solna, 17165 Stockholm, Sweden.; ^3^ Unité Dynamique Structurale des Macromolécules, Institut Pasteur, 25 Rue du Docteur Roux, FR-75015 Paris, France.; ^4^ Centre National de la Recherche Scientifique, CNRS UMR3528, Biologie Structurale des Processus Cellulaires et Maladies Infectieuses, 25 Rue du Docteur Roux, FR-75015 Paris, France.

## Abstract

A diverse set of modulators, including stimulants and anesthetics, regulates ion channel function in our nervous system. However, structures of ligand-bound complexes can be difficult to capture by experimental methods, particularly when binding is dynamic. Here, we used computational methods and electrophysiology to identify a possible bound state of a modulatory stimulant derivative in a cryptic vestibular pocket of a mammalian serotonin-3 receptor. We first applied a molecular dynamics simulation–based goal-oriented adaptive sampling method to identify possible open-pocket conformations, followed by Boltzmann docking that combines traditional docking with Markov state modeling. Clustering and analysis of stability and accessibility of docked poses supported a preferred binding site; we further validated this site by mutagenesis and electrophysiology, suggesting a mechanism of potentiation by stabilizing intersubunit contacts. Given the pharmaceutical relevance of serotonin-3 receptors in emesis, psychiatric, and gastrointestinal diseases, characterizing relatively unexplored modulatory sites such as these could open valuable avenues to understanding conformational cycling and designing state-dependent drugs.

## Teaser

A cryptic pocket for stimulant modulation of 5-HT_3_ receptors is discovered by enhanced sampling molecular dynamics simulations.

## INTRODUCTION

**Fig. 1.**
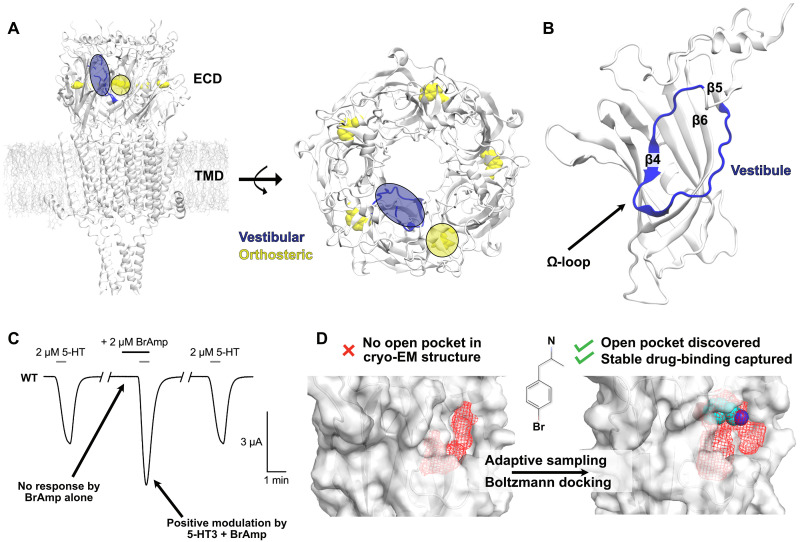
Overview of 5-HT_3A_R structure and pharmacology explored in this work. (**A**) Architecture of a representative 5-HT_3A_R, colored by subunit, viewed from the membrane plane (left) and from the extracellular side (right). 5-HT bound at the orthosteric site is represented in yellow. The vestibular site is colored blue. (**B**) ECD of a single 5-HT_3A_R subunit, with the omega-loop (Ω-loop) in blue. (**C**) Representative current trace from a 5-HT_3A_R–expressing *X. laevis* oocyte in the absence and presence of BrAmp during 5-HT (at EC_20_) pulses. (**D**) Pocket volumes at the 5-HT_3A_R vestibular site, generated in Fpocket (*73*), show a superficial cavity (volume, 524 Å^3^) in the experimental structure (left) but a clearly opened pocket (volume, 740 Å^3^) surrounding the docked pose of BrAmp (right), as found in our study. Pocket volumes are shown in red mesh, the ligand in van der Waals, and the receptor in gray surface representations.

Pentameric ligand-gated ion channels (pLGICs) play central roles in intercellular communication in the mammalian nervous system (*1*). In a classical example, neurotransmitters released at the synaptic cleft bind to corresponding pLGICs, contracting the extracellular domain (ECD) and opening a permeation pore in the transmembrane domain (TMD) to allow ions to cross the membrane for further signal transduction (Fig. 1A) (*2*). A diverse set of ligands, including anesthetics, neurosteroids, and lipids, can modulate these proteins via orthosteric (Fig. 1A, yellow) or various allosteric modulatory sites. For example, in serotonin [5-hydroxytryptamine (5-HT)] 3A receptors (5-HT_3A_Rs), antiemetic drugs such as palonosetron, used in the treatment of nausea and vomiting associated with radiation and chemotherapies, compete with 5-HT binding at the orthosteric site to inhibit channel function (*3*). Conversely, in γ-aminobutyric acid (GABA) type A receptors (GABA_A_Rs), classical benzodiazepine drugs that are used to treat epilepsy, anxiety, and insomnia bind sites distinct from that of GABA to modulate activation (*4*–*6*).

Whereas both the orthosteric neurotransmitter site and ECD benzodiazepine site are located at extracellular subunit interfaces, the ECD interior vestibule constitutes a relatively unexplored potential modulatory region (Fig. 1A, blue). Despite the recent determination of several ligand-bound experimental structures, none have yet captured binding at the vestibular site in eukaryotic pLGICs (*7*). In contrast, several x-ray structures of prokaryotic pLGICs do resolve ligands in this region, including the positive modulators 4-bromocinnamate [Protein Data Bank (PDB) ID: 6FLI] and 4-bromoamphetamine (BrAmp; PDB ID: 9EWL) bound to a channel derived from an endosymbiont of the tubeworm *Tevnia jerichonana* (sTeLIC) (Fig. 1C) (*8*, *9*). Similarly, in the *Erwinia chrysanthemi* and *Gloeobacter violaceus* ligand-gated ion channels (ELIC and GLIC), the benzodiazepine flurazepam (PDB ID: 2YOE, chain C) (*10*) and various modulatory carboxylates (PDB IDs: 6HJZ, 4QH1, and 6HPP), respectively, were found to bind in this location (fig. S1) (*11*–*13*). In these cases, ligands were identified between the β4, β5, and β6 strands, in a vestibular cavity defined by the so-called omega-loop (Fig. 1B).

In an effort to investigate a role for the ECD vestibular site in ligand binding and modulation of eukaryotic pLGICs, Brams and colleagues (*7*) performed a systematic analysis using available experimental structures. As demonstrated in their study, this cavity is occluded in experimental structures of most eukaryotic pLGICs, including α2/3 nicotinic acetylcholine receptor subunits (*14*, *15*) and α1/3 glycine receptor subunits (*16*). The vestibular cavity appears to be more accessible in 5-HT_3A_Rs (*17*); cysteine-scanning mutagenesis of human 5-HT_3A_Rs indicated this region to be sensitive to modulation by covalent labeling (*7*). However, the 5-HT_3A_R omega-loop is not conserved with those of prokaryotic channels known to bind vestibular ligands (fig. S1), and direct evidence for drug modulation via this region in a eukaryotic system remains lacking.

Molecular dynamics (MD)–based techniques offer complementary approaches to explore binding sites not readily apparent in experimental structures (*18*–*21*). Here, we applied a goal-oriented adaptive sampling method (*22*) to explore regions of conformational space relevant to the potential opening of this pocket in the ECD vestibule of a mammalian 5-HT_3A_R (Fig. 1, C and D). Then, to find plausible ligand-binding poses, we performed ensemble docking of BrAmp resulting in a total of 1.5 million docked poses and reweighted docking scores by Boltzmann-distribution probabilities of each protein conformation occurring, as derived from Markov state model (MSM) analysis of our trajectories. Following clustering of the top 100 docked poses, we then performed replicate unbiased MD simulations of representative complexes in two force fields to estimate ligand stability and screened the most stable complexes for pocket accessibility to the aqueous environment. For one relatively stable and accessible site, mutations predicted to disrupt BrAmp binding and/or modulation were validated by electrophysiology recordings in *Xenopus laevis* oocytes and provided a mechanistic rationale for the modulatory stabilization of an activated state.

## RESULTS

### Evidence for 5-HT_3A_R potentiation from functional but not structural data

In exploring potential vestibular pLGIC modulators, we noted that ligands previously crystallized in the vestibular site of the prokaryotic channel sTeLIC, i.e., 4-bromocinnamate, 4-bromophenethylamine, and BrAmp (*8*, *9*), are structurally similar to amphetamine psychostimulants. We tested the effect of BrAmp on 5-HT_3A_Rs expressed in *X. laevis* oocytes and found that low micromolar concentrations of the drug enhanced submaximal [2 μM, 20% maximal effective concentration (EC_20_)] 5-HT currents (Fig. 1C), with even greater apparent potency than in the bacterial channel (*9*). In the absence of 5-HT, BrAmp did not directly activate the receptor at up to 200 μM, suggesting that it acts not at the orthosteric 5-HT site but rather via an allosteric modulatory site (Fig. 1C and fig. S2). However, we found that the vestibular site shown to bind BrAmp in sTeLIC was relatively superficial in experimental 5-HT_3A_R structures, even in activated states presumed to be stabilized by positive allosteric modulators (PDB IDs: 6DG8 and 4PIR) (Fig. 1D and fig. S3) (*7*, *9*, *23*). Solvent-accessible volume was limited in buried regions of this pocket; although smaller dicarboxylates could fit (based on structural alignment with GLIC structures), ligands on the scale of BrAmp or larger (4-bromocinnamate and flurazepam superimposed from complexes with sTeLIC and ELIC) clashed with 5-HT_3A_R omega-loop side chains (fig. S1).

To more thoroughly investigate potential binding in this site, we performed molecular docking around the omega-loop of an activated 5-HT_3A_R structure and subjected this complex to unrestrained MD simulations. The modulator deviated rapidly (within 20 ns) and markedly [>15-Å root mean square deviation (RMSD)] from the docked pose and sampled increasingly distant poses over time (fig. S4). If the 5-HT_3A_R vestibular site does mediate BrAmp modulation, then it would appear to involve a cryptic pocket, i.e., a binding site that is intrinsically transient or, otherwise, not readily apparent in experimental structures (*18*). We also ran 25 replicate unrestrained simulations in the absence of modulator, totaling 1 μs, but observed limited flexibility in the omega-loop (root mean square fluctuation < 1.8 Å; fig. S5) relative to other regions, indicating that the conformational transitions are slow and that enhanced sampling methods would be required to capture an alternative open state of this putative binding site.

### An adaptive sampling and docking-based workflow

**Fig. 2.**
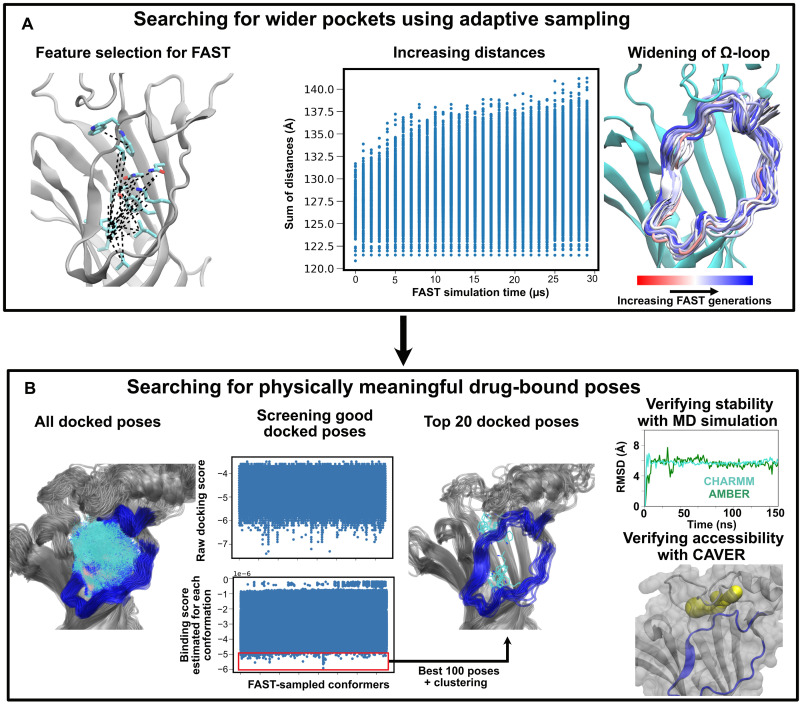
A two-phase computational workflow developed in this study to model first pocket opening and then ligand binding. (**A**) Left: In FAST sampling, starting points for successive generations were selected on the basis of pairwise distances (dashed lines) between residues (cyan) in the omega-loop (Ω-loop) and neighboring β strands. Center: These distances increased in successive FAST generations, each containing 1 μs of MD simulations data. Right: FAST sampling was also associated with progressive widening of the omega-loop, as depicted by representative snapshots colored by generation (red to blue). (**B**) Left: FAST-generated conformers were subjected to molecular docking with BrAmp. Center: docked scores were then re-weighted by the stationary probability of the corresponding protein conformation, determined by MSM analysis. The 100 best poses (red box) were then clustered on the basis of RMSD of the ligand atoms with a cutoff of 2.5 Å and screened down to 20 representative poses. Right: These poses were then used for further MD simulations and accessibility analysis.

We next applied an adaptive sampling method, fluctuation amplification of specific traits (FAST) (*22*), to launch simulations from the closed-pocket 5-HT_3A_R structure and maximize the chance of discovering cryptic pockets that may harbor ligand binding sites (see Materials and Methods for details) (Fig. 2A). Within 30 simulation generations, corresponding to 30 μs of total simulation, we observed an increase in pairwise distances between residues in the omega-loop and neighboring β-strand walls, associated with a widening of the omega-loop as well as more modest increases in pocket volume and solvent-accessible surface area (Fig. 2A and fig. S6). Further characterization of the energetic landscape using time-structure–independent components analysis (tICA) in pyEMMA showed three metastable states (fig. S7). The experimental starting structure projected to the lowest energy well, as expected. Representative frames projecting to the other two wells exhibited modest variations in and around the omega-loop, which could not be clearly distinguished by visual inspection (fig. S7).

To assess ligand binding to these explored conformations, we next performed ensemble docking of BrAmp to all FAST-sampled MD trajectories, a total of 30-μs simulations (Fig. 2B). Although a docking score can indicate the likelihood of binding a particular pocket conformation, its contribution to the actual free energy of binding will be low if this pocket conformation has a low probability of occurring. Hence, following the work of Hart and colleagues (*24*), we re-weighted the docking scores according to the equilibrium probabilities of the corresponding protein conformations based on a MSM of the FAST-sampled trajectories (Fig. 2B). Variations on this approach, termed MSM docking or PopShift, have been shown to improve activity predictions for small molecules in targets such as Temoneira (TEM) β-lactamase and myosin motor domains (*24*–*26*).

From the 100 best Boltzmann-docked BrAmp complexes, we reduced redundancy using RMSD-based clustering, resulting in 20 representative putative poses (Fig. 2B). We then tested their stability by performing unbiased MD simulations. Because small-molecule parameters can be sensitive to the choice of force fields, we performed three replicate simulations of each system in both CHARMM36m (*27*–*30*) and AMBER (*31*, *32*), resulting in 20 systems × 3 replicates × 2 force fields = 120 total 150-ns trajectories (fig. S8).

### A stable and accessible ligand binding site

**Fig. 3.**
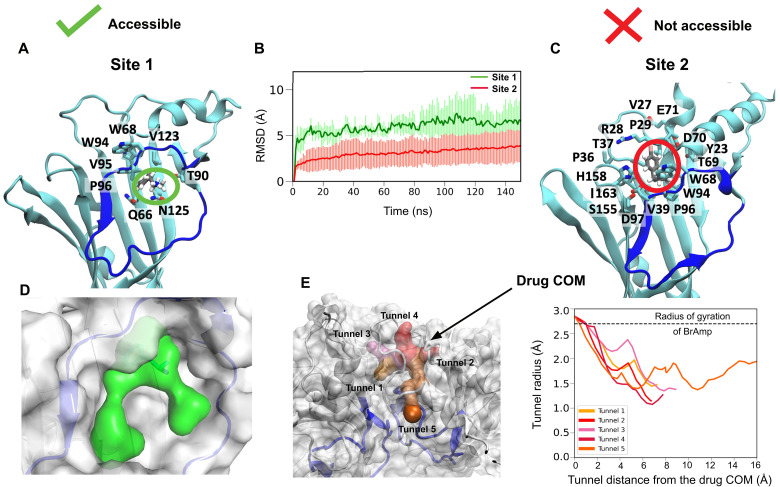
Comparison of stable sites captured by MD simulations of Boltzmann docked poses. (**A**) Interacting residues (cyan; >50% contact probability in MD simulations) surrounding the ligand (gray) in site 1. For clarity, hydrogens are hidden in protein side chains, and the omega-loop (Ω-loop) backbone is shown in blue. (**B**) Ligand stability in sites 1 (green) and 2 (red), as represented by RMSD of BrAmp from its initial docked pose during MD simulations, averaged across all stable replicates. Shaded regions indicate SDs. (**C**) Interacting residues surrounding the ligand in site 2, depicted as in (A). This site is located peripheral to the central vestibule. (**D**) Ligand pocket in site 1 (green) as modeled in Fpocket (*73*) at the endpoint of a representative 150-ns MD simulation, originally launched from pose 20 (fig. S8). The pocket is clearly accessible to solvent. (**E**) Accessibility analysis of site 2, as modeled in CAVER (*34*) at the endpoint of a representative 150-ns MD simulation, originally launched from pose 12 (fig. S8). Molecular representations (left) and tunnel profiles (right) show that all pathways (shades of red) to the ligand center of mass (COM) are restricted to ≤1.5-Å radius.

For closer analysis, we selected systems in which the ligand remained relatively stable (<15-Å center-of-mass RMSD) throughout at least five of its six replicate simulations. In the six systems that passed this filtering (fig. S8), we further analyzed the contact frequency of the ligand with each receptor residue (fig. S9). One of these systems (pose 20) positioned the ligand between the β2, β4, and β6 strands (site 1; Fig. 3, A and B). In the remaining five relatively stable systems (poses 8, 12, 13, 16, and 18), the ligand occupied a distinct region (site 2; Fig. 3, B and C) defined by the β1, β2, β4, and β8 strands. The ligand in site 1 reoriented early in the simulation to a converged pose ~6-Å RMSD from the initial docked pose (Fig. 3, A and B), suggesting a more optimized protein conformation than was initially sampled and/or that the presence of ligand induces a distinctive metastable complex.

Although even more stable than site 1, poses in site 2 were buried in an enclosed cavity, substantially shielded from solvent by the outermost ECD (Fig. 3, B and C). Computational docking and simulations might retain a ligand in a pocket inaccessible from the solvent, despite binding in such a site being physically implausible. Notably, in a previously curated dataset of experimental structures containing cryptic pockets (*19*, *33*), binding pockets were clearly solvent accessible in the majority (10 of 13) of bound complexes for which both apo and holo experimental structures were available (fig. S10). In pockets from this structural subset that were not obviously open to solvent, pathway analysis in the CAVER software suite (*34*) showed them to be accessible via tunnels at least 2 Å in radius (fig. S11). We therefore analyzed the accessibility of both putative sites in the 5-HT_3A_R using CAVER (Fig. 2B). In site 1, the ligand amino group was directly exposed to solvent, with the pocket clearly accessible to the aqueous medium (Fig. 3D). However, in site 2, accessibility analysis showed no pathways for ligand entry greater than 1.5-Å radius (Fig. 3E and movie S1). Accordingly, we proceeded to focus on site 1 as a plausible region for ligand binding and modulation.

### Structural features and functional validation of a vestibular modulatory site

**Fig. 4.**
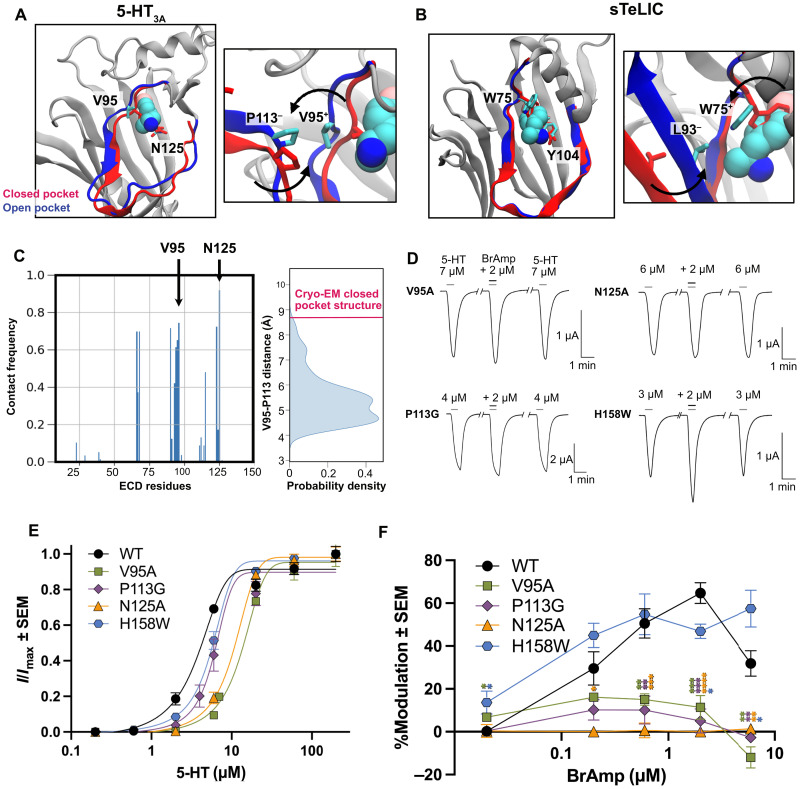
Structural features and functional validation of a vestibular modulatory site of 5-HT_3A_R. (**A**) Left: Remodeling of the β4 strand in open-pocket (blue) versus closed-pocket (red) models of the 5-HT_3A_R enables stable binding of BrAmp (cyan spheres) between residues V95 and N125. Right: V95 interacts with P113 on the complementary subunit upon transitioning from closed to open pocket conformations. (**B**) View as in (A) of the bacterial homolog sTeLIC, showing structures in the absence (PDB ID: 9EX6, red) and the presence of BrAmp (blue; PDB ID: 9EWL) (*9*). sTeLIC residues W75, Y104, and L93, corresponding to 5-HT_3A_R V95, N125, and P113 respectively, are shown. (**C**) Left: Frequency of contacts between BrAmp in site 1 and individual residues of the 5-HT_3A_R ECD in MD simulations. A contact is counted if two atoms of the ligand and receptor are within 4 Å of one another. Right: Probability distribution of the minimum distance between the side-chain atoms of V95 and P113 on neighboring subunits, calculated over all stable MD simulation replicates associated with site 1. The distance captured in the cryo-EM structure (PDB ID: 6DG8) is highlighted red. (**D**) Representative current traces from four mutant 5-HT_3A_R–expressing *Xenopus* oocytes in the absence and presence of BrAmp during EC_20_ 5-HT pulses. (**E**) Concentration-response curves for 5-HT activation of WT and mutant 5-HT_3A_Rs expressed in *Xenopus* oocytes. Each point indicates mean ± SEM current normalized to the maximum recorded in a given oocyte (*I*/*I*_max_). *n* ≥ 4 biological replicates; individual data points are reported in table S1. (**F**) Concentration-response plot displaying the mean percent modulation ± SEM of WT, V95A, P113G, N125A, and H158W 5-HT_3A_Rs by various concentrations of BrAmp. Asterisks indicate significance relative to WT (*P ≤ 0.05; **P ≤ 0.01; ***P ≤ 0.001; ****P ≤ 0.0001). *n* ≥ 4 biological replicates; individual data points, including *P* values, are reported in table S2.

The putative 5-HT_3A_R vestibular site contained several hydrophobic residues (β2-W68, β4-W94, and β6-V123) lining the inner wall, making van der Waals contacts with the bromine and aromatic groups of the ligand (Fig. 3, A and B). The mouth of the putative site contained more polar residues (β2-Q66, β3-T90, and β6-N125), interacting with the ligand amino group. The binding pose was similar to that observed in x-ray structures of this and related ligands in sTeLIC (Fig. 4, A and B). In comparing the experimental structure to the putative bound state derived from our simulations, the most marked change in the omega-loop was an outward shift and rotation of the β4 strand, relieving prospective clashes of the ligand with residues including β4-V95 (Figs. 1C and 4A). Local resolution is relatively poor in this region of β4 in multiple activated 5-HT_3A_R cryo–electron microscopy (cryo-EM) structures (fig. S12), indicating that it might be flexible and possibly sample such alternative conformations. This transition moved V95 roughly 4 Å toward the subunit interface, bringing it into direct contact with residue P113 on β5 of the complementary subunit (Fig. 4, A and C). A similar mechanism of side chain rotation and intersubunit interaction was also captured in structures of sTeLIC (Fig. 4B) (*9*), suggesting a generalizable model for pocket opening and ligand binding. The inward-facing end of the omega-loop was previously proposed to promote closed-to-open transitions of the 5-HT_3A_R by a similar mechanism, during which β4-F103 moves out of the vestibular site to interact with β6-P128 on the complementary subunit (*35*). These effects are consistent with the general contraction of the upper ECD associated with pLGIC activation (*2*).

To functionally validate our putative ligand complex, we first engineered mutations at V95 and N125, two of the most frequent amino acid contacts in simulations of this ligand pose (Fig. 4, A, C, and D). Substituting alanine at either of these positions weakened apparent 5-HT sensitivity, increasing the agonist EC_50_ roughly twofold in 5-HT_3A_R expressing oocytes (Fig. 4E and table S1). Moreover, at 5-HT concentrations giving equivalent levels of activation, either substitution significantly reduced potentiation by 600 nM to 6 μM BrAmp, rendering the receptor largely insensitive to the modulator (Fig. 4, D and F, and table S2). We further hypothesized that drug displacement of V95 toward P113 in the complementary subunit would promote interfacial contraction, thereby facilitating channel activation (Fig. 4, A, C, and D). In support of this model, substituting glycine at P113 markedly suppressed potentiation by BrAmp, similar to its direct contacts in the vestibular site (Fig. 4, D and F, and tables S1 and S2). In contrast, substituting tryptophan at H158, a residue that substantially contacts BrAmp in site 2 (>50% contact frequency in four of the five simulation systems; fig. S9), did not significantly alter channel modulation at 200 nM to 6 μM BrAmp, consistent with this site being less functionally relevant (Fig. 4, D and F, and tables S1 and S2). Thus, functional recordings of site-directed mutants were in agreement with computational measurements of stability and accessibility in predicting a vestibular potentiating mechanism in the 5-HT_3A_R.

## DISCUSSION

The resolution revolution has increasingly enabled single-particle cryo-EM reconstructions of previously inaccessible systems, including complex membrane proteins such as pLGICs (*36*). While these advances have tremendous potential to launch an era of structure-based drug design, relatively few structures have been determined in the presence of prospective drugs. Visualizing drug binding at an atomic level, including the ligand and protein interactions and geometries, can support the design of pharmaceuticals that mimic the chemical structure, binding mode, and functional effect of known modulators. A persistent challenge in the field is that binding pockets may not be readily apparent in unliganded experimental structures, due to their transient nature combined with induced or selected fit binding mechanisms. Even when it is possible to resolve flexibility, for example, by cryo-EM, protein conformations compatible with binding might have too low probability of being detected. Therefore, to exploit the full potential of structural data, complementary types of methods are needed.

MD simulation and machine learning–based techniques offer notable opportunities to explore cryptic or otherwise experimentally obscure sites (*18*–*21*). In MD simulations, a major challenge is the timescale required to transition a cryptic pocket from a closed to open state. Popular methods, in addition to FAST (*22*), include sampling water interfaces through scaled Hamiltonians, in which nonbonded interactions of solvent molecules with protein atoms are progressively scaled, shifting the water properties toward more ligand-like behavior to promote cryptic-pocket opening (*18*). A general challenge to MD-based methods is the need for computing resources and a system-specific simulation setup, particularly for larger protein complexes. To address this challenge, one recent study demonstrated the power of stochastic subsampling of multiple sequence alignment depth in AlphaFold2 combined with Markov state modeling to accelerate the discovery of cryptic pockets (*20*, *37*). Another study showed the effectiveness of training graph neural networks based on a large MD dataset to find cryptic pockets (*19*). The FAST approach used here has been successfully applied to capturing pockets in Ebola viral protein 35 (*38*), severe acute respiratory syndrome coronavirus 2 (*39*), and the epidermal growth factor receptor (*40*), a notable diversity of targets.

Computational methods are inherently models offering predictions, and, to validate these predictions, we have used electrophysiology functional assays. This work demonstrates a pipeline that was able to reveal a functionally validated vestibular binding of an amphetamine derivative in a eukaryotic pLGIC. Amphetamines are widely used to treat diseases such as attention deficit hyperactivity disorder, narcolepsy, and irritable bowel syndrome (*41*). However, several adverse side effects such as anorexia, weight loss, insomnia, and dependence have been reported (*41*). The amphetamine-5-HT_3A_R complex proposed in this study could provide a starting point for designing analogous compounds to circumvent such side effects. BrAmp produces toxicity in brainstem neurons (*42*) and depletes brain 5-HT with a longer half-life than other amphetamines (*43*); although its action has been largely attributed to transporters and metabotropic receptors for 5-HT (*44*), exploration of its effects on ionotropic receptors could provide fresh insight into the neuropharmacology of this and related compounds. A similar scaffold is featured in the contemporary recreational psychedelic 4-bromo-2,5-dimethoxyphenethylamine (*45*). It remains to be seen whether other eukaryotic channels such as glycine, acetylcholine, or GABA_A_Rs also contain modulatory sites in the vestibular region; application of our method to these systems could substantially enrich the development of pLGIC pharmacology.

Our results raise several mechanistic questions regarding vestibular modulation of 5-HT_3A_Rs. First, because we simulated pocket opening in the absence of a modulator, our study cannot conclusively distinguish whether drug binding occurs via a conformational selection, induced fit, or a combined mechanism. Metadynamics analysis could provide relevant insight, for instance, by using our bound pose as a starting model and pocket opening/closing as well as drug binding/unbinding as reaction coordinates. Second, despite the fact that BrAmp alone is incapable of activating 5-HT_3A_R even at relatively high concentrations (Fig. 1C and fig. S2), our study does not rule out the possibility that the ligand can also occupy the orthosteric 5-HT–binding site. Although beyond the scope of the current work, it will be interesting to see whether future structural or functional experiments succeed in validating either binding pose.

Third, although our electrophysiology results showed that the ligand acts as a positive modulator, it remains unclear how ligand binding at the vestibular site may influence allosteric coupling between orthosteric 5-HT binding and pore opening. Because we were mainly interested in sampling the vestibular pocket in the ECD, located around 45 Å from the TMD, we mildly restrained the M2 helix of the TMD to prevent commonly observed pore-collapsing events of pLGICs in MD simulations (*46*). Dynamic allosteric coupling could, in principle, be investigated using MD-based methods such as Markov state modeling or metadynamics, with our putative complex as a starting point and using polarizable or other force fields that may better represent the hydration of the pore (*47*). Given past evidence for cross-talk between the orthosteric and vestibular binding site in the bacterial channel GLIC (*11*–*13*) and of vestibular mechanisms of zinc modulation in the anion-conducting glycine receptor (*48*), future work may elucidate a similar mechanism of allosteric modulation in 5-HT_3A_Rs.

Overall, our results show how MD simulation–based methods can be used to sample previously unseen or cryptic binding pockets in cryo-EM structures and identify stable drug binding that can be validated with experimental assays. This appears to be a promising approach for sampling structural transitions in homologous pLGIC binding sites, including pharmaceutically relevant targets like the 5-HT_3A_R.

## MATERIALS AND METHODS

### System preparations

Simulations were initiated from the 5-HT–bound open-state cryo-EM structure of 5-HT_3A_R (PDB ID: 6DG8) (*23*). The structure was embedded a symmetric membrane that approximates neuronal plasma membrane composition (*49*): 44.4% cholesterol, 22.2% 1-palmitoyl-2-oleoyl-*sn*-glycero-3-phosphocholine, 22.2% 1-palmitoyl-2-oleoyl-*sn*-glycero-3-phosphoethanolamine, 10% 1-palmitoyl-2-oleoyl-*sn*-glycero-3-phospho-l-serine, and 1.1% phosphatidylinositol 4,5-bisphosphate. The system was built using the Membrane Builder module of CHARMM-GUI (*50*) by solvating with transferable intermolecular potential with 3 points (TIP3P) water (*51*) and neutralizing in 0.15 M NaCl to generate systems containing 300,000 atoms, with dimensions of 130 Å by 130 Å by 200 Å. To allow for better sampling, 25 independent replicas were built by randomly configuring initial lipid placement around the protein using the Membrane mixer plugin in VMD (*52*, *53*). With initially diverse lipid placements, there could be metastable substates separated by slow lipid motions that are bypassed by the Membrane mixer; however, we applied mild restraints in the TMD (see below) expected to mitigate any allosteric effects of protein-lipid interactions.

The systems were energy minimized and then relaxed in simulations at constant pressure (1 bar) and temperature (310 K) for 30 ns, during which the position restraints on the protein and ligands were gradually released. The restraints were used as recommended by CHARMM-GUI. Then, production runs were performed with a mild position restraint of 50 kJ mol^−1^ nm^−2^ on the backbone atoms of the pore-facing residues at the M2 helix of the TMD to prevent the commonly known pore-collapsing events of ligand-gated ion channels in MD simulations (*46*). Also, we maintained a mild flat-bottom restraint of 20 kJ mol^−1^ nm^−2^ between the atoms of each 5-HT molecule and residues in its binding site to prevent spontaneous unbinding of agonist as we have observed in previous simulations of related channels (*6*).

### Adaptive sampling

To explore possible pocket openings at the vestibular site, we applied a goal-oriented adaptive sampling method, called FAST (*22*). Briefly, the method runs successive swarms of simulations where the starting points for each swarm are chosen from the set of all previously discovered conformations based on a reward function. This function balances (i) preferentially simulating structures with maximum pairwise distances (Fig. 2A) to encourage the omega-loop to adapt a more open conformation that may harbor cryptic pockets and with (ii) a broad exploration of conformational space. The pairwise distances were chosen from the residues located at the beta strands of ECD to the same at the omega-loop, resulting in a total of 125 pairs, 25 per monomer. The selection of residue pairs was based on previously reported cryo-EM structures of closed- and open-pocket states of the BrAmp-sensitive bacterial channel sTeLIC (Fig. 4B). We defined distances on the basis of the nearest atoms in each pair, rather than, e.g., Cα atoms, given that at least one critical determinant of BrAmp modulation in sTeLIC appeared to be the side-chain orientation of a key vestibular residue (*9*). Volume-based metrics in the vestibular pocket were less consistent in our hands, resulting in variable continuity (fig. S3). The broad exploration phase was implemented by favoring states that are poorly sampled compared to other states, based on the RMSD of the ECD residues. During FAST, we performed 30 generations of simulations with 25 simulations per generation and 40 ns per simulation, totaling 30 μs. Because no biasing force is applied to any individual simulation, the final dataset can be used to build a MSM to extract the proper thermodynamics and kinetics (*54*–*56*), as detailed below.

### Markov state modeling

We used our trajectory dataset from FAST to construct a MSM using pyEmma (*57*) by first featurizing the trajectory dataset using the 125 residue-residue distance pairs used for FAST sampling, as described above. We used pairwise distances as an intuitive metric for expansion of a large superficial pocket and on the basis of previous experience using equivalent features to build MSMs in this protein family (*58*, *59*). The conformational space was then discretized into 1000 microstates using *k*-means clustering. Then, a transition probability matrix (TPM) was constructed by evaluating the probability of transitioning between each microstate within a lag time, τ. To choose an adequate lag time to construct a TPM that ensures Markovian behavior, multiple TPMs were first created using multiple maximum-likelihood MSMs with different lag times. The implied timescales were evaluated for each of these transition matrices, which largely converged at τ = 5 ns (fig. S13). Thus, we built our final TPM using a maximum likelihood MSM with a lag time of 5 ns. This final TPM is symmetrized using a maximum likelihood approach to ensure detailed balance (*57*).

### Metastable state analysis

We visualized the free-energy landscape using tICA to reduce the dimensionality of the feature space *X*(*t*) to the eigenvectors of an autocovariance matrix, ⟨*X*(*t*)*X*^T^(*t* + τ)⟩, with a lag time τ = 1 ns (*60*–*62*). To choose an optimal number of tICA eigenvectors for building the associated MSM, we calculated variational approach for Markov processes (VAMP-2) scores and found that the first four tICA eigenvectors were sufficient (fig. S14) (*63*). Other hyperparameters such as the number of clusters and lag time were as described in the previous section. Free energies of each frame were projected onto the first two time-structure independent component (tIC) vectors.

### MD simulations

MD simulations in this study were performed using GROMACS-2023 (*64*) using CHARMM36m (*27*) and CHARMM36 (*65*) force field parameters for proteins and lipids, respectively. The force field parameters for the ligands were generated using the CHARMM General Force Field (*28*–*30*). Cation-π interaction-specific non-bonded fix (NBFIX) parameters were used to maintain appropriate ligand-protein interactions at the aromatic cage, located at the binding sites (*66*). Bonded and short-range nonbonded interactions were calculated every 2 fs, and periodic boundary conditions were used in all three dimensions. The particle mesh Ewald method (*67*) was used to calculate long-range electrostatic interactions with a grid spacing below 0.1 nm^−3^. A force-based smoothing function was used for pairwise nonbonded interactions at 1 nm with a cutoff of 1.2 nm. Pairs of atoms whose interactions were evaluated were searched and updated every 20 steps. A cutoff of 1.2 nm was applied to search for the interacting atom pairs. Constant pressure was maintained at 1 bar using the Parrinello-Rahman barostat (*68*), and temperature was kept at 300 K with the v-rescale thermostat (*69*).

### Molecular docking

All the FAST sampled conformations were used for molecular docking of BrAmp using AutoDock Vina (*70*). A grid box of dimensions of 24 Å by 22 Å by 25 Å at the vestibular site was used for docking, outputting the best docking scored pose for each protein conformation.

### Statistical analysis

System visualization and analysis were carried out using VMD and PyMOL (*53*, *71*). The “measure cluster” module implemented in VMD (*72*) was used for clustering analysis. A hydrogen bond was counted between an electronegative atom with a hydrogen atom (H) covalently bound to it [the donor (D)] and another electronegative atom [the acceptor (A)], provided that the distance D-A was less than 3 Å and the angle D-H-A was more than 120°.

### Expression in oocytes

The gene encoding the mouse 5-HT_3A_R was inserted into the pBK-CMV expression vector (Agilent). The desired mutations were generated via site-directed mutagenesis using Phusion High-Fidelity DNA Polymerase (Thermo Fisher Scientific) along with commercial mutagenic primers [Integrated DNA Technologies (IDT)]. The polymerase chain reaction product was digested overnight with Dpn I at 37°C and subsequently transformed into XL1-Blue Supercompetent cells (Agilent). Verification of the mutant cDNAs was performed via Sanger sequencing (Eurofins Genomics), followed by large-scale purification using a HiSpeed Plasmid Midi Kit (QIAGEN). Oocytes from female *X. laevis* frogs (Ecocyte Bioscience) were injected via the animal pole with 6 ng/32.2 nl of cDNA using a Nanoject II microinjector (Drummond Scientific). After injection, the oocytes were incubated for a period ranging from 3 to 9 days at 13°C in modified Barth’s solution [88 mM NaCl, 1 mM KCl, 2.4 mM NaHCO_3_, 0.91 mM CaCl_2_, 0.82 mM MgSO_4_, 0.33 mM Ca(NO_3_)_2_, 10 mM Hepes, 0.5 mM theophylline, 0.1 mM G418, 17 mM streptomycin, penicillin (10,000 U/liter), and 2 mM sodium pyruvate, adjusted to pH 7.5], before conducting two-electrode voltage-clamp (TEVC) recordings.

### Electrophysiology

For TEVC recordings, glass electrodes filled with 3 M KCl (5 to 50 megohms) were used to establish a voltage clamp on the oocyte membrane at −70 mV, using an OC-725C voltage clamp (Warner Instruments). The oocytes were continuously perfused with a running buffer (123 mM NaCl, 2 mM KCl, and 2 mM MgSO_4_, adjusted to pH 7.5), at a flow rate of ~1 ml/min. Data sampling and digitization were carried out with a Digidata 1440A (Molecular Devices) in combination with Clampex software (Axon Instruments). The currents were filtered at a frequency of 1 kHz and analyzed using Clampfit (Axon Instruments).

For each BrAmp recording, a 1 M stock solution of BrAmp was prepared in dimethyl sulfoxide. The wash period between each 30-s application was 10 min. After each BrAmp-containing application, the two subsequent applications were solely running buffer with ∼EC_20_ 5-HT to verify resensitization. To investigate whether BrAmp could directly activate 5-HT_3A_R, oocytes were washed with BrAmp for 1 min and then immediately exchanged to a ∼EC_20_ 5-HT–containing buffer with the same concentration of BrAmp for 30 s. All statistical analyses pertaining to electrophysiology data were conducted using Prism 10.0.3 (GraphPad Software). The analyzed data were obtained from groups comprising at least five independent experiments. The results are presented as means ± SEM and were evaluated using unpaired Student’s *t* tests, with significant effects set at *P* < 0.05. Concentration-response curves were derived using the following equation: *Y* = {*R*_basal_ + [(*R*_max_ − *R*_basal_)/(1 + 10^(logEC50−logX)*nH^)]}, where *Y* is the current response to a given concentration of 5-HT, *R*_max_ is the maximum response, *R*_basal_ is the baseline response, *X* is the 5-HT concentration, EC_50_ is the concentration that elicits 50% of the maximum response, and *n*_H_ is the Hill coefficient. BrAmp effects were calculated as % modulation = [(*R*_A_ − *R*_0_)/*R*_0_] ∗ 100, where *R*_0_ is the current response to EC_20_ 5-HT and *R*_A_ is the subsequent response to EC_20_ 5-HT plus modulator.
