# Supplementary material for: Discovering cryptic pocket opening and binding of a stimulant derivative in a vestibular site of the 5-HT3A receptor
Source: Sci Adv. 2025 Apr 11;11(15):eadr0797. doi: 10.1126/sciadv.adr0797 (PMC11988449; doi:10.1126/sciadv.adr0797)
Supplement: Supplementary file 1 — Figs. S1 to S14 Tables S1 and S2 Legend for movie S1 [file sciadv.adr0797_sm.pdf]

Science Advances
AAAS

Supplementary Materials for

Discovering cryptic pocket opening and binding of a stimulant derivative in a vestibular site of the 5-HT_{3A} receptor

Nandan Haloi *et al.*

Corresponding author: Rebecca J. Howard, rebecca.howard@scilifelab.se; Erik Lindahl, erik.lindahl@dbb.su.se

Sci. Adv. **11**, eadr0797 (2025)
DOI: 10.1126/sciadv.adr0797

The PDF file includes:

Figs. S1 to S14
Tables S1 and S2
Legend for movie S1

Other Supplementary Material for this manuscript includes the following:

Movie S1

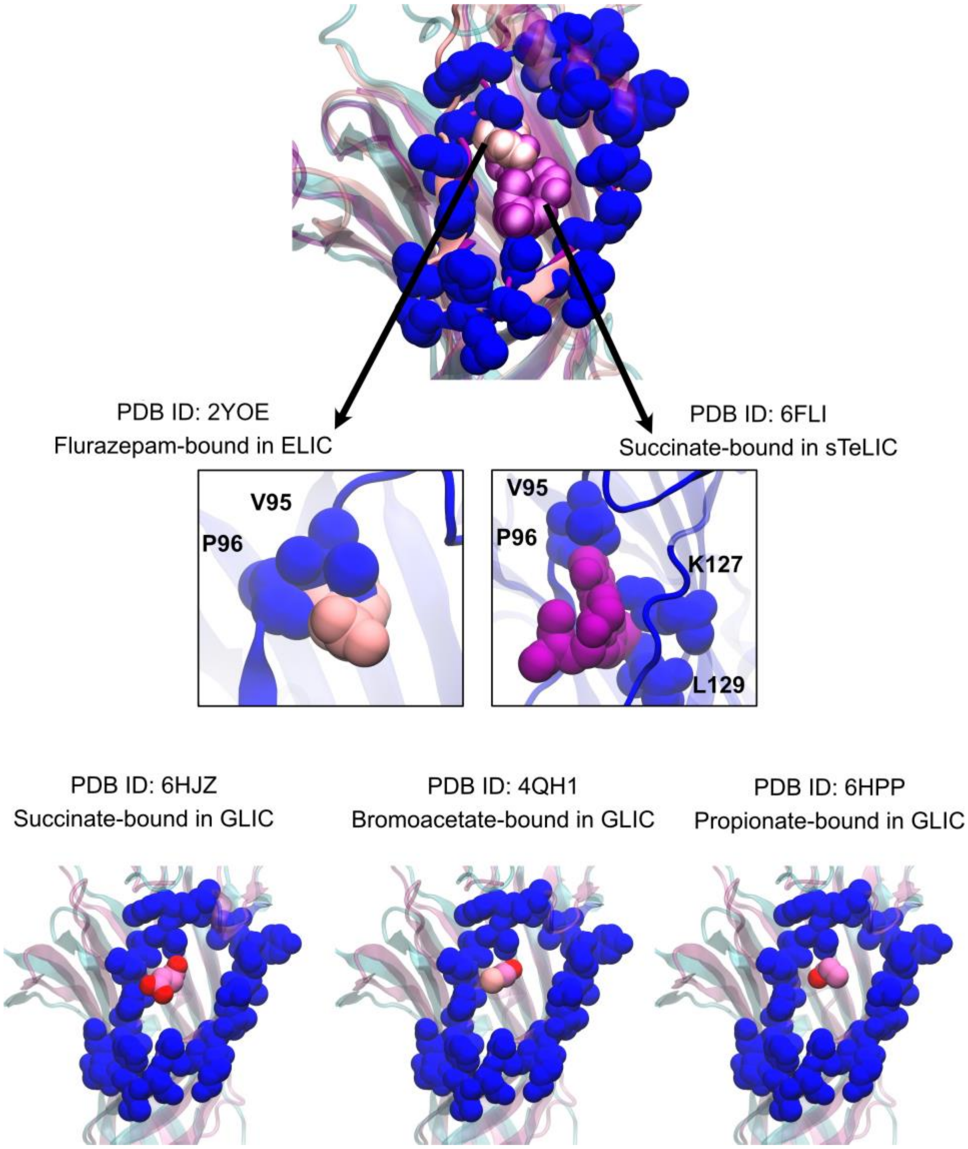

Fig. S1. Structural alignment of putative vestibular sites in various bacterial pLGICs (lavender) with the 5-HT$_{3A}$R (blue). In ELIC (pink), sTeLIC (magenta), and GLIC (lavender), spheres represent vestibule-bound carboxylates; in the 5-HT$_{3A}$R (PDB ID: 6DG8), spheres represent amino-acid side chains in the Ω-loop.

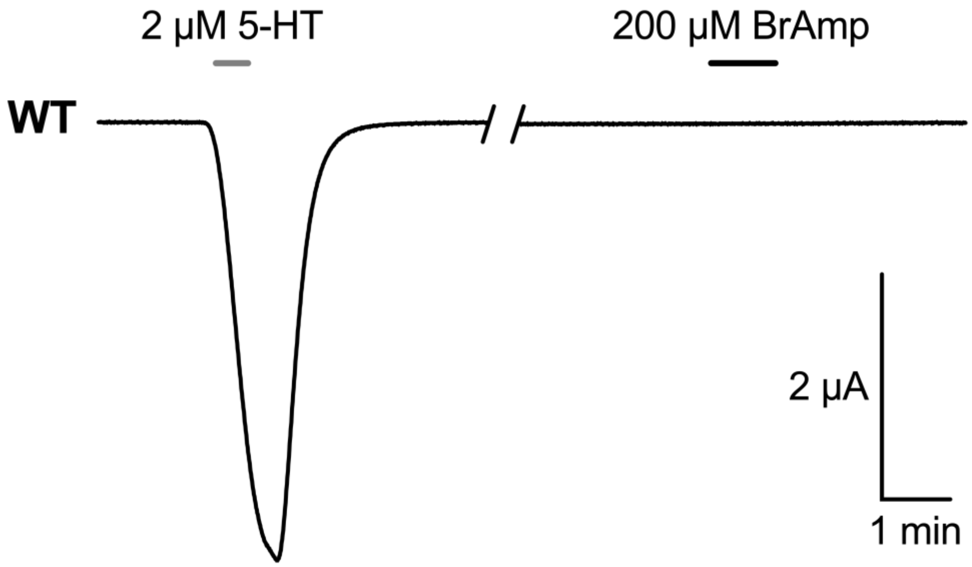

Fig. S2. Sample oocyte electrophysiology trace. The trace showing a lack of direct activation by BrAmp up to 200 µM.

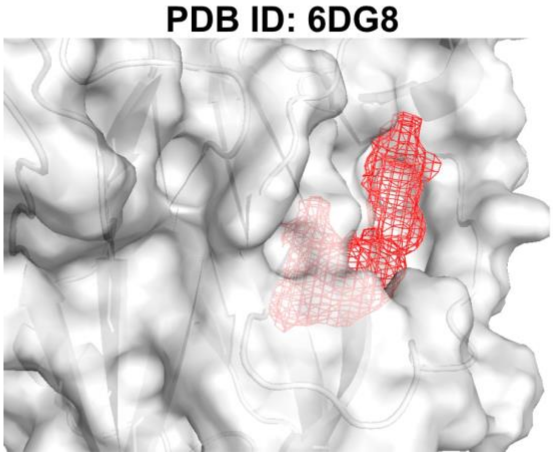

Fig. S3. Constricted pockets at the 5-HT$_{3A}$R vestibular site. Volumes generated in Fpocket (*23*) for two different activated experimental structures, PDB IDs: 6DG8 (524 Å^3) and 4PIR (408 Å^3), show no clear cavity for drug binding, particularly in more deeply buried regions of the vestibular pocket (upper left).

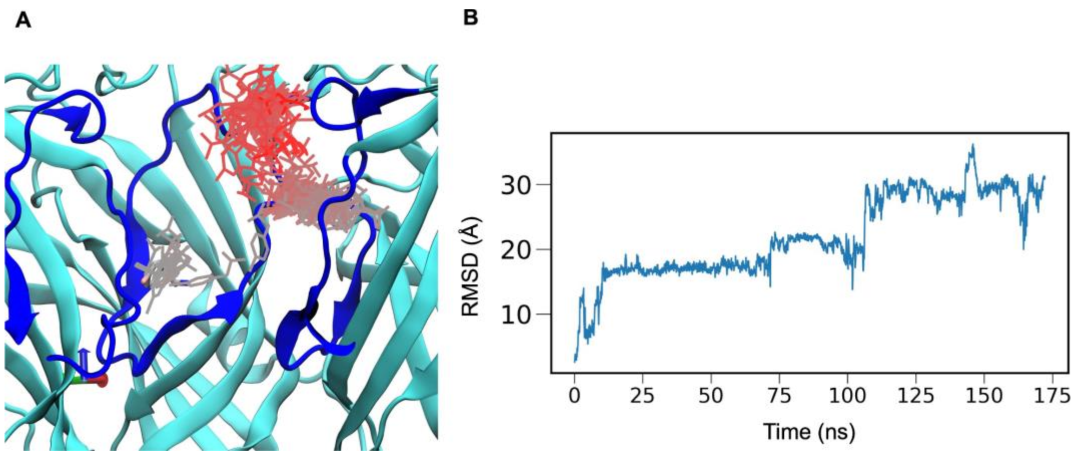

Fig. S4. Docking of BrAmp to an experimental 5-HT$_{3A}$R structure. A) Docking BrAmp to an activated-state 5-HT$_{3A}$R (PDB ID: 6DG8) (*24*) did not produce stable binding, as illustrated by the time evolution of the ligand during simulation, colored by frame (white to red). B) RMSD of the ligand with respect to its original docked position during MD simulation.

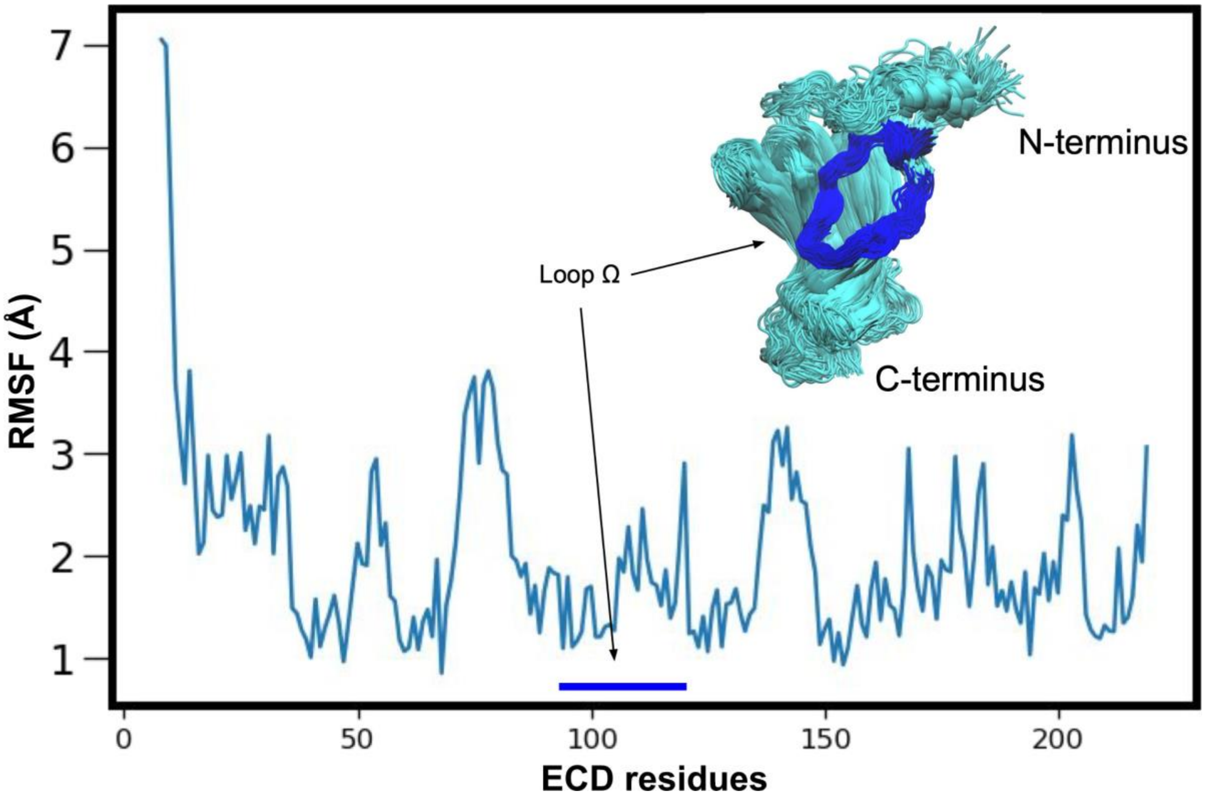

Fig. S5. Fluctuation in the ECD during enhanced sampling. RMSF of ECD residues over the first generation of FAST simulations (25 replicates, 1 μs total simulation time). Inset shows sampled ECD conformations as cyan ribbons, with the Ω-loop in blue.

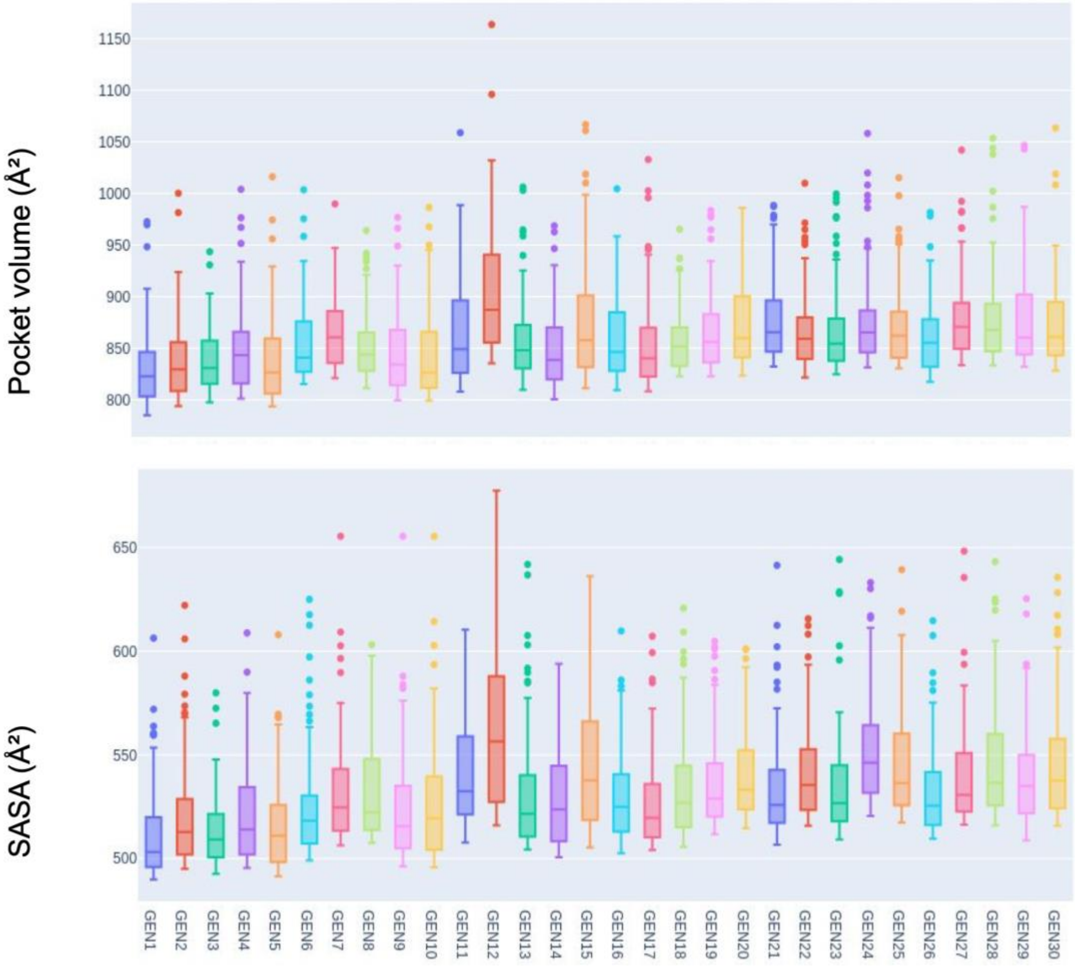

Fig. S6. Vestibular site metrics during enhanced sampling. Box plots of pocket volumes (*above*) and solvent-accessible surface areas (SASA, *below*) at the vestibular site, calculated using CAVER (*34*) for the highest 100 values in each FAST generation.

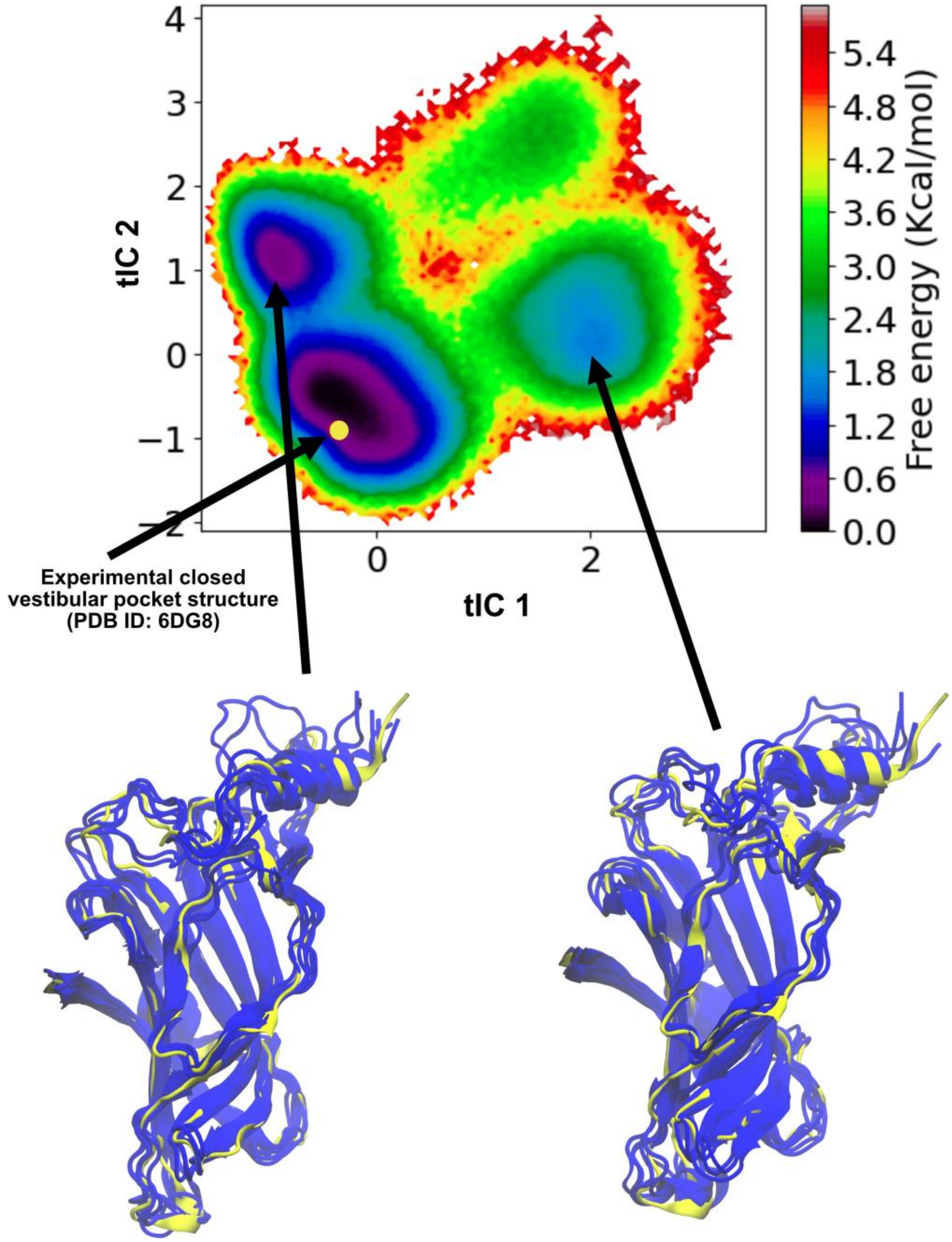

Fig. S7. Structural characteristics of metastable states captured in the MSM-derived free-energy landscape. *Above*, simulation frames from FAST sampling, projected onto the top two tICA eigenvectors and colored by free energy according to scalebar at right. Experimental starting structure (PDB ID: 6DG8, yellow) projects to the deepest well in the landscape. *Below*, superpositions of single-subunit ECDs from snapshots projecting to two other free-energy wells (blue) with the experimental starting structure (yellow), showing broadly distributed conformational sampling.

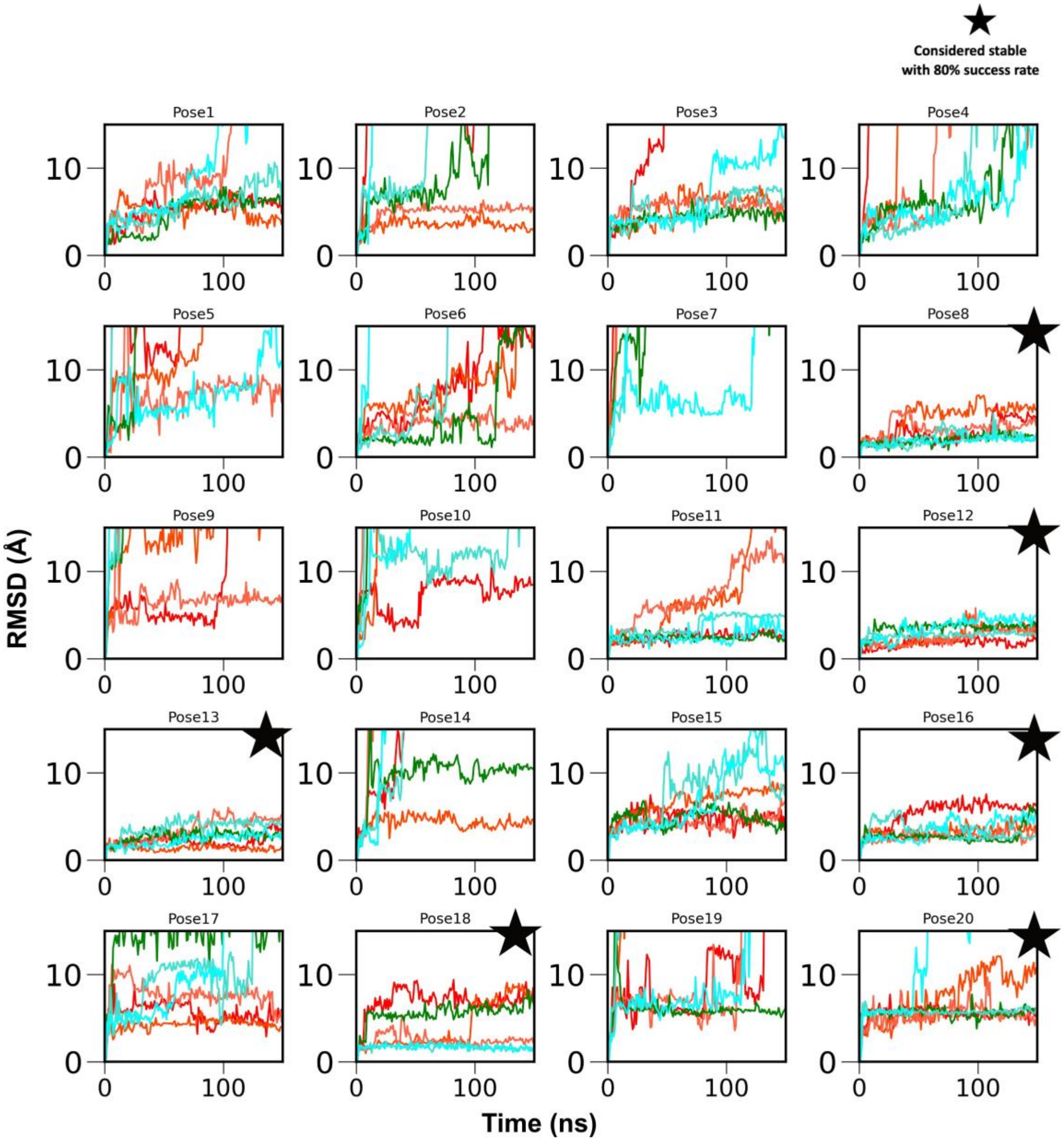

Fig. S8. Stability of BrAmp in simulations from docked poses. Each plot indicates RMSDs of BrAmp from a particular docked pose during three replicate MD simulations each in CHARMM36 (shades of blue) and AMBER (shades of red). Stars indicate systems selected for further analysis due to remaining within 15 Å RMSD in at least 5 replicates.

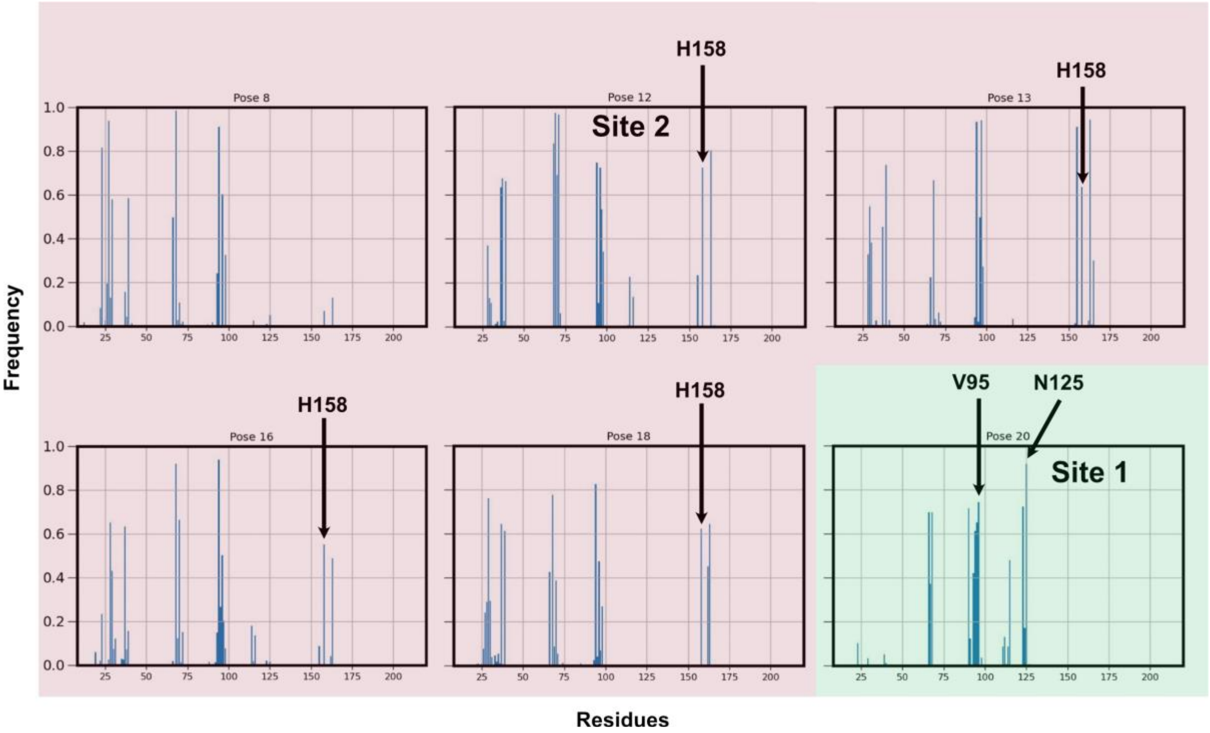

Fig. S9. Frequency of contacts of BrAmp to individual residues of the 5-HT$_{3A}$R in stable (<15-Å RMSD) MD simulations of selected poses (fig. S8). A contact is counted if two atoms of the ligand and receptor are within 4 Å. Green and red shading indicates categorization as sites 1 or 2 respectively, based on patterns of contacting residues.

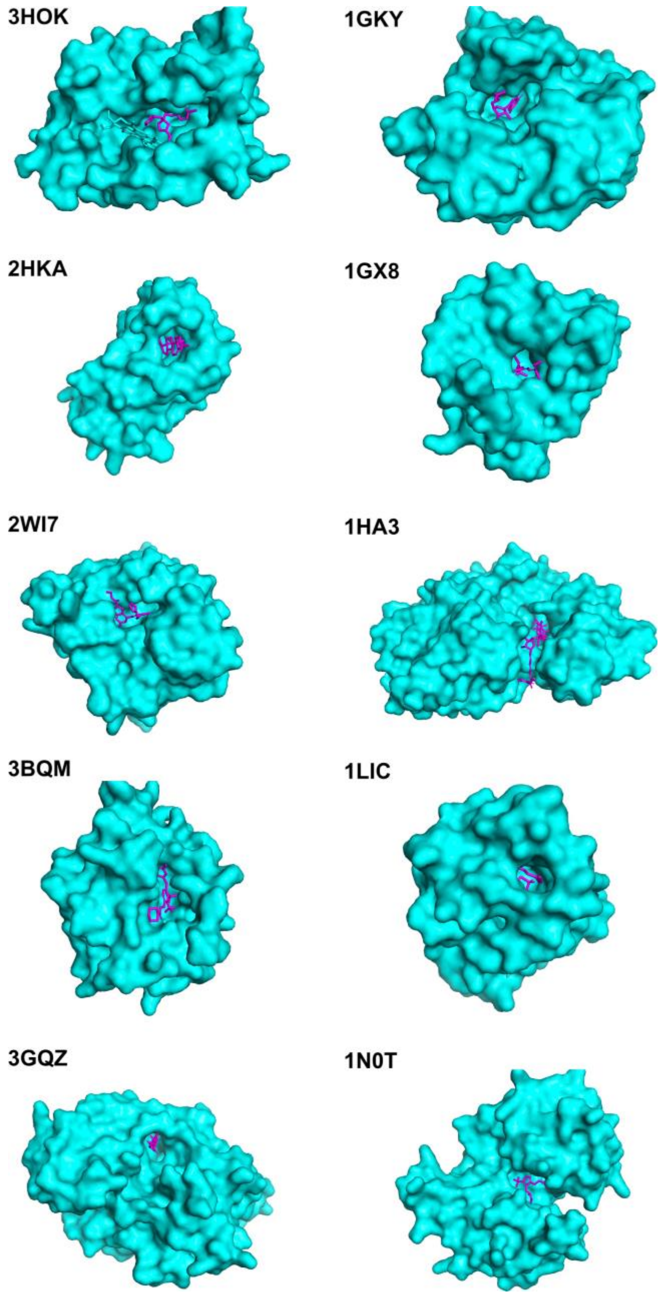

Fig. S10. Cryptic pockets with clear accessibility from the protein surface to ligand sites in the bound state. Systems are from a previously curated dataset of cryptic pockets (*19*, *35*), with protein surfaces in cyan and ligands as purple sticks.

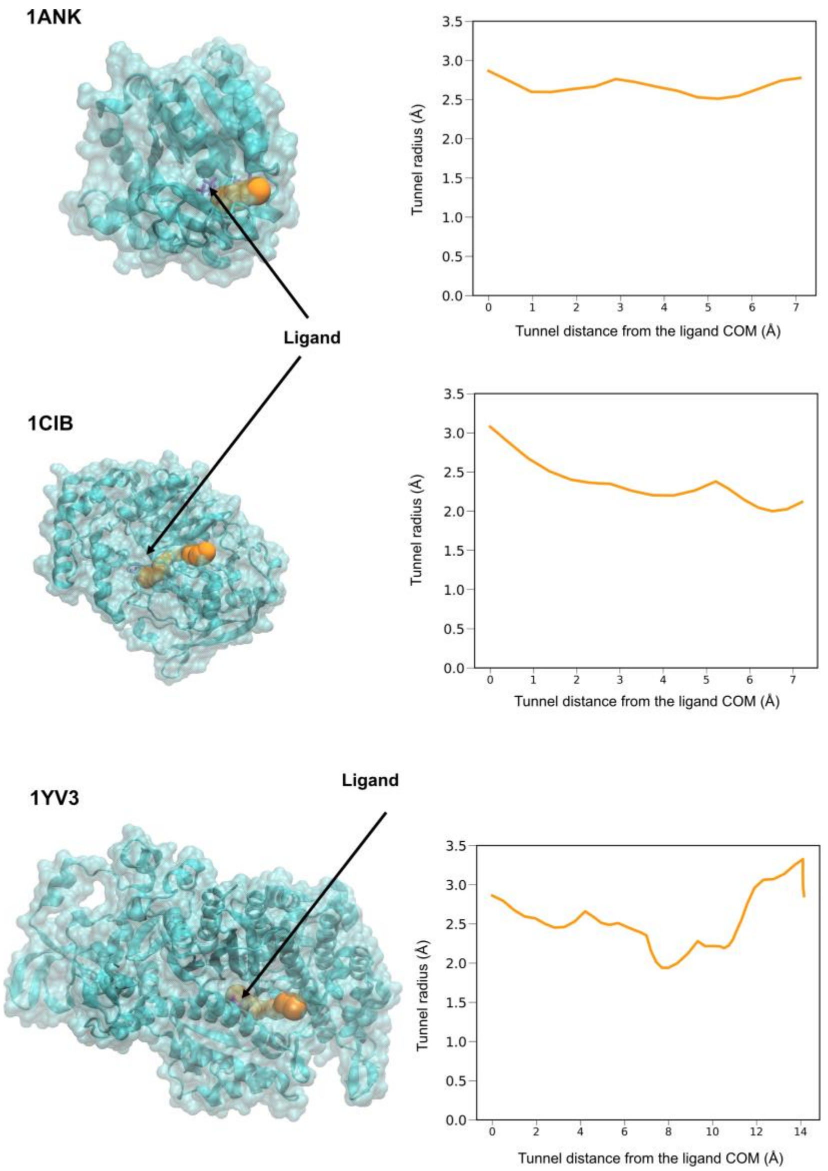

Fig. S11. Cryptic pockets with >2 Å-radius accessibility tunnels to ligand sites in the bound state. Systems are from a previously curated dataset of cryptic pockets (*19*, *35*). *Left*, holo experimental structures (green) showing accessibility tunnels (orange) to binding pockets from the protein surface, calculated in CAVER. *Right*, tunnel radius plots show pathways at least 2 Å in radius to the ligand center of mass (COM).

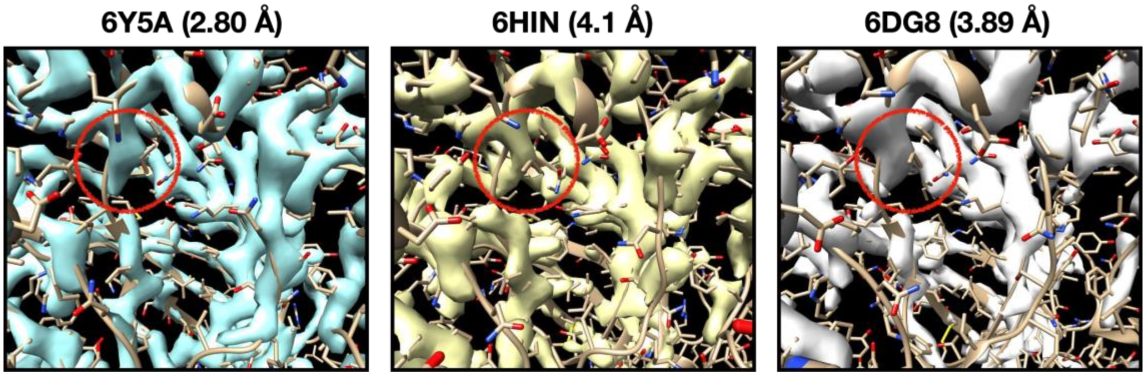

Fig. S12. Limited Ω-loop resolution in 5-HT$_{3A}$R structures. Cryo-EM densities (surfaces) and corresponding models (sticks, colored by heteroatom) for three experimental structures of the mouse 5-HT$_{3A}$R, labeled by PDB ID and overall resolution. Red circles highlight densities around residue V95, where backbone continuity is typically disrupted.

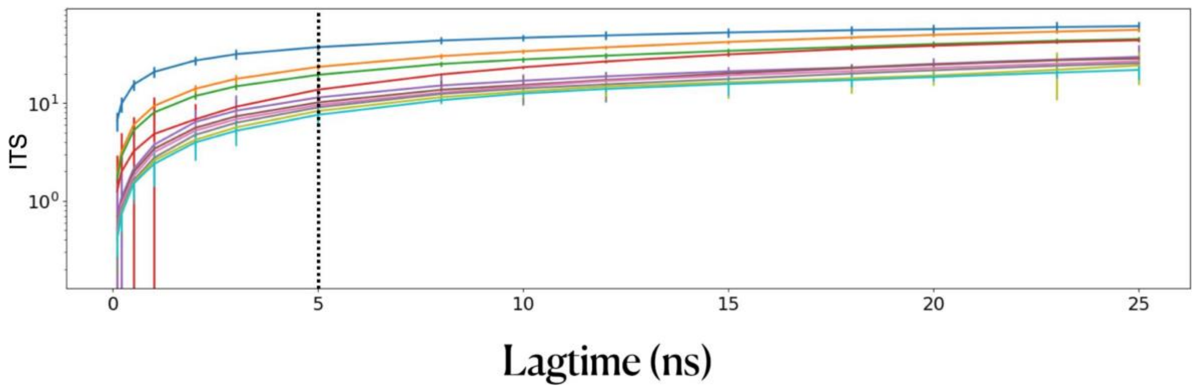

Fig. S13. Convergence of implied timescales of slowest MSM transitions over increasing lagtimes. Implied timescales (ITS) plotted for the 10 slowest processes (blue to green) using lagtimes up to 25 ns in Markov state modeling of FAST sampling trajectories. Error bars indicate uncertainty evaluated using a Bayesian estimated MSM. A lagtime of 5 ns (dotted line) was chosen for MSM construction.

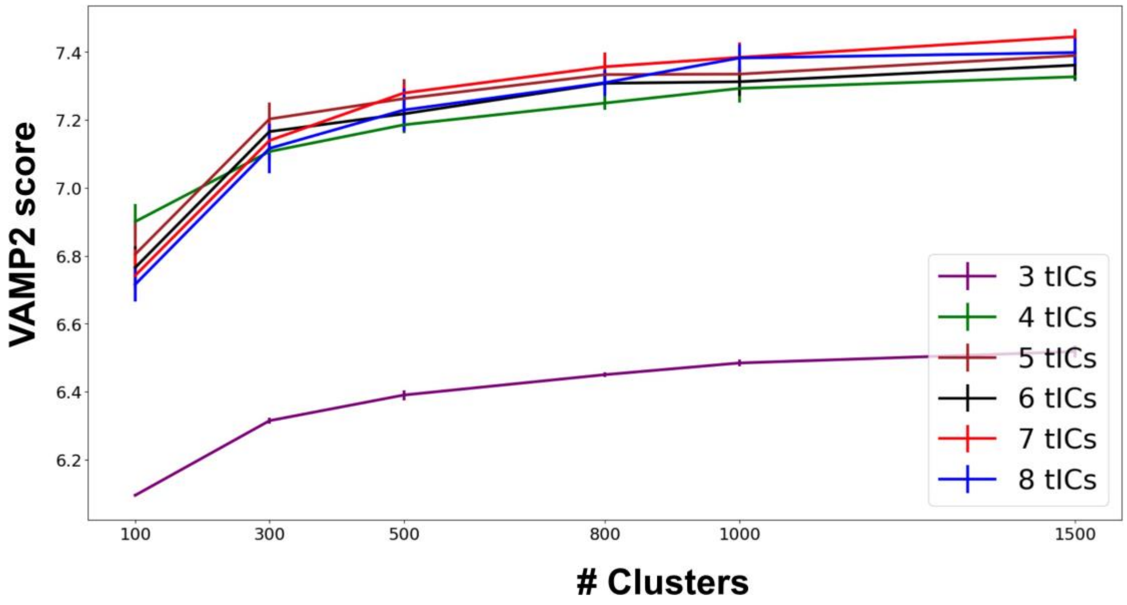

Fig. S14. Validation of tICA eigenvectors. Cross-validated VAMP-2 scores (*64*) plotted over increasing numbers of clusters, using 3–8 tICA eigenvectors (purple to blue according to legend at lower right) to discretize the conformational space. Each value indicates mean score ± standard deviation based on 5 iterations of the *k*-means clustering algorithm.

WT

Measurements	REF 200 μM	20 nM	200 nM	600 nM	2 μM	6 μM	20 μM	60 μM	200 μM
Current-1	-1.867	0	0	-0.006	-0.165	-1.329	-1.72	-1.859	-1.939
Current-2	-7.001	0	0	-0.092	-2.115	-4.607	-5.507	-6.546	-7.605
Current-3	-2.59	0	0	-0.013	-0.454	-1.604	-1.948	-2.171	-2.59
Current-4	-0.505	0	0	-0.003	-0.095	-0.385	-0.443	-0.484	-0.503
Current-5	-3.098	0	0	-0.006	-0.531	-2.121	-2.35	-2.567	-2.621

Condition (5-HT)	Control (5-HT)	1	2	3	4	5
20 nM	200 μM	0	0	0	0	0
200 nM	200 μM	0	0	0	0	0
600 nM	200 μM	0.0032	0.0131	0.005	0.0059	0.001936733376
2 μM	200 μM	0.0884	0.3021	0.1753	0.1881	0.1714009038
6 μM	200 μM	0.7118	0.658	0.6193	0.7624	0.6846352485
20 μM	200 μM	0.9213	0.7866	0.7521	0.8772	0.7585539057
60 μM	200 μM	0.9957	0.935	0.8382	0.9584	0.8285990962
200 μM	200 μM	1.0386	1.0863	1	0.996	0.8460296966

	Mean	Std Err	N
20 nM	0	0	5
200 nM	0	0	5
600 nM	0.00585026483	0.001950901322	5
2 μM	0.1850572133	0.03415938207	5
6 μM	0.6872405055	0.02421212984	5
20 μM	0.8191542309	0.03392942108	5
60 μM	0.9111926423	0.03323208252	5
200 μM	0.9933814464	0.04027023371	5

V95A

Measurements	REF 200 μM	20 nM	200 nM	2 μM	6 μM	7 μM	20 μM	60 μM	200 μM
Current-1	-1.2	0	0	-0.009	-0.125	-0.2495	-0.896	-1.074	-1.141
Current-2	-6.039	0	0	-0.012	-0.675	-1.212	-4.021	-4.669	-4.701
Current-3	-4.181	0	0	-0.006	-0.318	-0.7275	-2.856	-3.151	-3.807
Current-4	-0.782	0	0	-0.002	-0.073	-0.1513	-0.565	-0.759	-0.935
Current-5	-1.502	0	0	-0.007	-0.18	-0.2829	-1.125	-1.53	-1.536

Condition (5-HT)	Control (5-HT)	1	2	3	4	5
20 nM	200 μM	0	0	0	0	0
200 nM	200 μM	0	0	0	0	0
2 μM	200 μM	0.0075	0.002	0.0014	0.0026	0.00466045273
6 μM	200 μM	0.1042	0.1118	0.0761	0.0934	0.119840213
7 μM	200 μM	0.2079	0.2007	0.174	0.1935	0.1883
20 μM	200 μM	0.7467	0.6658	0.6831	0.7225	0.7490013316
60 μM	200 μM	0.895	0.7731	0.7536	0.9706	1.018641811
200 μM	200 μM	0.9508	0.7784	0.9105	1.1957	1.022636485

	Mean	Std Err	N
20 nM	0	0	5
200 nM	0	0	5
2 μM	0.003628028965	0.001111435863	5
6 μM	0.101037819	0.007621168009	5
7 μM	0.1929061655	0.005778862675	5
20 μM	0.7134206554	0.01678959772	5
60 μM	0.8822037495	0.05244628514	5
200 μM	0.9716219694	0.0686561959	5

P113G

Measurements	REF 200 μM	200 nM	2 μM	4 μM	6 μM	20 μM	60 μM	200 μM
Current-1	-2.9	0	-0.112	-	-1.114	-1.58	-2.5	-2.866
Current-2	-0.31	0	-0.02	-0.046	-0.078	-0.229	-0.267	-0.3
Current-3	-3.4	0	-0.153	-0.707	-2.24	-3.046	-3.165	-3.29
Current-4	-1.276	0	-0.015	-0.085	-0.275	-0.876	-1.047	-1.203
Current-5	-1.978	0	-0.049	-0.7	-1.135	-1.746	-1.846	-1.893

Condition (5-HT)	Control (5-HT)	1	2	3	4	5
200 nM	200 μM	0	0	0	0	0
2 μM	200 μM	0.0386	0.0645	0.045	0.0118	0.02477249747
4 μM	200 μM	-	0.1484	0.2079	0.0666	0.353892821
6 μM	200 μM	0.3841	0.2516	0.6588	0.2155	0.5738
20 μM	200 μM	0.5448	0.7387	0.8959	0.6865	0.8827
60 μM	200 μM	0.8621	0.8613	0.9309	0.8205	0.9333
200 μM	200 μM	0.9883	0.9677	0.9676	0.9428	0.957

	Mean	Std Err	N
200 nM	0.0000	0.0000	5
2 μM	0.0369	0.0090	5
4 μM	0.1942	0.0606	4
6 μM	0.4168	0.0872	5
20 μM	0.7497	0.0652	5
60 μM	0.8816	0.0219	5
200 μM	0.9647	0.0075	5

N125A

Measurements	REF 200 μM	20 nM	200 nM	2 μM	6 μM	20 μM	60 μM	200 μM
Current-1	-0.128	0	0	0	-0.012	-0.128	-0.144	-0.153
Current-2	-2.627	0	0	-0.007	-0.446	-2.395	-2.764	-2.807
Current-3	-0.27	0	0	-0.002	-0.08	-0.231	-0.253	-0.265
Current-4	-0.119	0	0	0	-0.029	-0.109	-0.114	-0.119
Current-5	-0.178	0	0	-0.003	-0.035	-0.165	-0.172	-0.174

Condition (5-HT)	Control (5-HT)	1	2	3	4	5
20 nM	200 μM	0	0	0	0	0
200 nM	200 μM	0	0	0	0	0
2 μM	200 μM	0	0.0027	0.0074	0	0.01685393258
6 μM	200 μM	0.0938	0.1698	0.2963	0.2437	0.1966
20 μM	200 μM	1	0.9117	0.8556	0.916	0.927
60 μM	200 μM	1.125	1.0522	0.937	0.958	0.9663
200 μM	200 μM	1.1953	1.0685	0.9815	1	0.9775

	Mean	Std Err	N
20 nM	0.0000	0.0000	5
200 nM	0.0000	0.0000	5
2 μM	0.0054	0.0032	5
6 μM	0.2000	0.0342	5
20 μM	0.9220	0.0231	5
60 μM	1.0077	0.0353	5
200 μM	1.0446	0.0411	5

H158W

Measurements	REF 200 μM	20 nM	200 nM	600 nM	2 μM	6 μM	20 μM	60 μM	200 μM
Current-1	-0.3032	0	0	-0.0038	-0.0268	-0.1629	-0.2801	-0.2958	-0.3024
Current-2	-1.076	0	0	-0.01	-0.119	-0.562	-0.974	-0.997	-1.021
Current-3	-0.3578	0	0	-0.007	-0.0381	-0.1782	-0.335	-0.3564	-0.3598
Current-4	-1.521	0	0	-0.0015	-0.0353	-0.5115	-1.2121	-1.442	-1.498
Current-5	-0.582	0	0	-0.012	-0.054	-0.373	-0.523	-0.564	-0.579

Condition (5-HT)	Control (5-HT)	1	2	3	4	5
20 nM	200 μM	0	0	0	0	0
200 nM	200 μM	0	0	0	0	0
600 nM	200 μM	0.0125	0.0093	0.0196	0.001	0.0206185567
2 μM	200 μM	0.0884	0.1106	0.1065	0.0232	0.09278350515
6 μM	200 μM	0.5373	0.5223	0.498	0.3363	0.6408934708
20 μM	200 μM	0.9238	0.9052	0.9363	0.7969	0.8986254296
60 μM	200 μM	0.9756	0.9266	0.9961	0.9481	0.9690721649
200 μM	200 μM	0.9974	0.9489	1.0056	0.9849	0.9948453608

	Mean	Std Err	N
20 nM	0.0000	0.0000	5
200 nM	0.0000	0.0000	5
600 nM	0.0126	0.0036	5
2 μM	0.0843	0.0158	5
6 μM	0.5070	0.0491	5
20 μM	0.8922	0.0247	5
60 μM	0.9631	0.0119	5
200 μM	0.9863	0.0099	5

Table S1. 5-HT concentration responses from individual oocytes expressing WT, V95A, P113G, N125A, or H158W 5-HT_{3A}Rs, summarized in Fig. 4E.

Modulation

WT - BrAmp

Measurements	REF 2 μM 5-HT	20 nM BrAmp	REF 2 μM 5-HT	200 nM BrAmp	REF 2 μM 5-HT	600 nM BrAmp	REF 2 μM 5-HT	2 μM BrAmp	REF 2 μM 5-HT	6 μM BrAmp
Current-1	-1.87	-1.861	1.962	-2.701	-0.077	-0.114	-1.205	-2.2	-4.261	-5.002
Current-2	-1.503	-1.5040	-2.359	-2.737	-0.702	-1.162	-1.358	-2.084	-4.344	-5.463
Current-3	-1.265	-1.275	-2.56	-2.988	-1.214	-1.798	-1.669	-2.596	-1.151	-1.692
Current-4	-1.41	-1.49	-0.081	-0.133	-0.632	-0.976	-1.16	-1.892	-1	-1.003
Current-5	-1.43	-1.397	-0.102	-0.118	-0.698	-1.164	-1.4	-2.214	-0.712	-0.886
Current-6	-1.879	-1.834	-1.563	-1.987	-1.29	-1.555	-1.164	-2.042	-2.548	-3.152

Condition (5-HT + BrAmp)	Control (5-HT)	1	2	3	4	5	6
2 μM + 20 nM	2 μM	-0.4813	0.0665	0.7905	5.6738	-2.3077	-2.394890899
2 μM + 200 nM	2 μM	37.6656	16.0237	16.7188	64.1975	15.6863	27.12731926
2 μM + 600 nM	2 μM	48.0519	65.5271	48.1054	54.4304	66.7622	20.54263566
2 μM + 2 μM	2 μM	82.5726	53.4610	55.5422	63.1034	58.1429	75.42955326
2 μM + 6 μM	2 μM	17.3903	25.7597	47.0026	52.8963	24.4382	24

	Mean	Std Err	N
**20 nM**	3.0000	0.8718981937	5
**200 nM**	0.7484	1.333758495	5
**600 nM**	30.0584	9.499533229	5
**2 μM**	56.5754	4.079802718	5
**6 μM**	62.5644	5.255242311	5

P-values

Symbol	Meaning
ns	P > 0.05
*	P ≤ 0.05
**	P ≤ 0.01
***	P ≤ 0.001
****	P ≤ 0.0001

V95A - BrAmp

Measurements	REF 7 μM 5-HT	20 nM BrAmp	REF 7 μM 5-HT	200 nM BrAmp	REF 7 μM 5-HT	600 nM BrAmp	REF 7 μM 5-HT	2 μM BrAmp	REF 7 μM 5-HT	6 μM BrAmp
Current-1	-1.252	-1.313	-0.713	-0.853	-0.865	-0.992	-1.404	-1.564	-0.848	-0.734
Current-2	-1.111	-1.2	-0.596	-0.65	-0.58	-0.612	-1.02	-1.029	-0.536	-0.424
Current-3	-0.098	-0.102	-0.073	-0.085	-0.078	-0.09	-0.059	-0.061	-0.101	-0.102
Current-4	-0.546	-0.6	-0.447	-0.53	-0.483	-0.565	-0.592	-0.782	-0.633	-0.48
Current-5	-0.688	-0.738	-0.256	-0.299	-0.592	-0.725	-0.248	-0.271	-0.797	-0.78

Condition (5-HT + BrAmp)	Control (5-HT)	1	2	3	4	5
7 μM + 20 nM	7 μM	4.8722	8.0108	4.0816	9.8901	7.2674
7 μM + 200 nM	7 μM	19.6353	9.0604	16.4384	18.5682	16.7969
7 μM + 600 nM	7 μM	14.6821	5.5172	15.3846	16.9772	22.4662
7 μM + 2 μM	7 μM	11.3960	0.8824	3.3898	32.0946	9.2742
7 μM + 6 μM	7 μM	-13.4434	-20.8955	0.9901	-24.1706	-2.1330

	Mean	Std Err	N
**20 nM**	6.8244	1.056793519	5
**200 nM**	16.0998	1.854007509	5
**600 nM**	15.0055	2.737018164	5
**2 μM**	11.4074	5.511663795	5
**6 μM**	-11.9305	4.977016343	5

V95A vs WT - BrAmp

	P-values
**20 nM**	0.0071
**200 nM**	0.1582
**600 nM**	0.0160
**2 μM**	<0.0001
**6 μM**	0.0004

P113G - BrAmp

Measurements	REF 4 μM 5-HT	20 nM BrAmp	REF 4 μM 5-HT	200 nM BrAmp	REF 4 μM 5-HT	600 nM BrAmp	REF 4 μM 5-HT	2 μM BrAmp	REF 4 μM 5-HT	6 μM BrAmp
Current-1	-2.254	-2.231	-0.204	-0.256	-0.482	-0.623	-0.2	-0.253	-1.302	-1.207
Current-2	-2.165	-2.273	-4.235	-4.839	-4.44	-4.917	-4.59	-4.377	-4.254	-4.157
Current-3	-1.458	-1.506	-4.287	-4.424	-4.942	-5.005	-4.152	-4.039	-4.442	-4.22
Current-4	-1.812	-1.697	-4.168	-4.076	-4.143	-3.793	-0.347	-0.357	-4.122	-3.937
Current-5	-2.435	-2.454	-0.345	-0.38	-0.336	-0.396	-0.367	-0.379	-0.35	-0.369

Condition (5-HT + BrAmp)	Control (5-HT)	1	2	3	4	5
4 μM + 20 nM	4 μM	-1.0204	4.9885	3.2922	-6.3466	0.7803
4 μM + 200 nM	4 μM	25.4902	14.2621	3.1957	-2.2073	10.1449
4 μM + 600 nM	4 μM	29.2531	10.7432	1.2748	-8.4480	17.8571
4 μM + 2 μM	4 μM	26.5000	-4.6405	-2.7216	2.8818	3.2698
4 μM + 6 μM	4 μM	-7.2965	-2.2802	-4.9977	-4.4881	5.4286

	Mean	Std Err	N
**20 nM**	0.3388	1.963139978	5
**200 nM**	10.1771	4.760736421	5
**600 nM**	10.1361	6.516649185	5
**2 μM**	5.0579	5.577974761	5
**6 μM**	-2.7268	2.189187236	5

P113G vs WT - BrAmp

	P-values
**20 nM**	0.9634
**200 nM**	0.0743
**600 nM**	0.0023
**2 μM**	<0.0001
**6 μM**	0.0007

N125A - BrAmp

Measurements	REF 6 μM 5-HT	20 nM BrAmp	REF 6 μM 5-HT	200 nM BrAmp	REF 6 μM 5-HT	600 nM BrAmp	REF 6 μM 5-HT	2 μM BrAmp	REF 6 μM 5-HT	6 μM BrAmp
Current-1	-0.58	-0.629	-1.27	-1.324	-0.369	-0.418	-1.973	-2.072	-1.613	-1.674
Current-2	1.807	-1.697	-1.63	-1.596	-1.05	-1.035	-2.071	-1.966	-1.749	-1.803
Current-3	-0.595	-0.558	-1.584	-1.578	-0.909	-0.922	-1.648	-1.667	-1.648	-1.638
Current-4	-1.895	-2.011	-1.288	-1.241	-0.521	-0.482	-0.253	-0.242	-1.921	-1.842
Current-5	1.807	-1.797	-1.05	-1.071	-0.501	-0.488	-0.507	-0.525	-1.736	-1.816

Condition (5-HT + BrAmp)	Control (5-HT)	1	2	3	4	5
6 μM + 20 nM	6 μM	8.4483	-6.0874	-6.2185	6.1214	-0.5534
6 μM + 200 nM	6 μM	4.2520	-2.0859	-0.3788	-3.6491	2.0000
6 μM + 600 nM	6 μM	13.2791	-1.4286	1.4301	-7.4856	-2.5948
6 μM + 2 μM	6 μM	5.0177	-5.0700	1.1529	-4.3478	3.5503
6 μM + 6 μM	6 μM	3.7818	3.0875	-0.6068	-4.1124	4.6083

	Mean	Std Err	N
**20 nM**	0.3421	3.035558114	5
**200 nM**	0.0276	1.412188554	5
**600 nM**	0.6401	3.472322277	5
**2 μM**	0.0606	2.045749025	5
**6 μM**	1.3517	1.631198051	5

N125A vs WT - BrAmp

	P-values
**20 nM**	0.9715
**200 nM**	0.0079
**600 nM**	0.0002
**2 μM**	<0.0001
**6 μM**	0.0013

H158W - BrAmp

Measurements	REF 3 μM 5-HT	20 nM BrAmp	REF 3 μM 5-HT	200 nM BrAmp	REF 3 μM 5-HT	600 nM BrAmp	REF 3 μM 5-HT	2 μM BrAmp	REF 3 μM 5-HT	6 μM BrAmp
Current-1	-2.975	-3.121	-0.0317	-0.0471	-0.0326	-0.0505	-0.024	-0.0346	-0.019	-0.0314
Current-2	-2.482	-2.5963	-0.0338	-0.0478	-0.0358	-0.0538	-0.0164	-0.0255	-0.0217	-0.0324
Current-3	-0.5118	-0.6458	-0.0631	-0.083	-0.0648	-0.0869	-0.0313	-0.0464	-0.2316	-0.4105
Current-4	-0.1236	-0.1468	-0.3848	-0.6097	-0.5439	-0.9784	-1.24	-1.75	-0.3491	-0.482

Condition (5-HT + BrAmp)	Control (5-HT)	1	2	3	4
3 μM + 20 nM	3 μM	4.9076	4.6052	26.1821	18.7702
3 μM + 200 nM	3 μM	48.5804	41.4201	31.5372	58.4459
3 μM + 600 nM	3 μM	54.9080	50.2793	34.1049	79.8860
3 μM + 2 μM	3 μM	44.1667	55.4878	48.2428	41.1290
3 μM + 6 μM	3 μM	65.2632	49.3088	77.2453	38.0693

	Mean	Std Err	N
**20 nM**	13.6163	5.3347	4
**200 nM**	44.9959	5.6839	4
**600 nM**	54.7946	9.4782	4
**2 μM**	47.2566	3.1067	4
**6 μM**	57.4716	8.6351	4

H158W vs WT - BrAmp

	P-values
**20 nM**	0.0198
**200 nM**	0.1875
**600 nM**	0.7204
**2 μM**	0.0274
**6 μM**	0.0341

Table S2. BrAmp modulation from individual oocytes expressing WT, V95A, P113G, N125A, or H158W 5-HT_{3A}Rs, summarized in Fig. 4F.

Movie S1. Accessibility tunnels to vestibular site 2, plotted in CAVER, mapped in 50 representative snapshots from simulations depicted in fig. S8. Accessibility to this site was tightly restricted throughout all simulations analyzed.
